# Facile Synthesis of β-Lactoglobulin-Functionalized Reduced Graphene Oxide and Trimetallic PtAuPd Nanocomposite for Electrochemical Sensing

**DOI:** 10.3390/nano8090724

**Published:** 2018-09-13

**Authors:** Bingkai Han, Meixin Pan, Jiexin Zhou, Yingying Wang, Zihua Wang, Jun Jiao, Cong Zhang, Qiang Chen

**Affiliations:** 1 The Key Laboratory of Bioactive Materials Ministry of Education, College of Life Science, Nankai University, Weijin Road No. 94, Tianjin 300071, China; hanbingkai@mail.nankai.edu.cn (B.H.); 2120171090@mail.nankai.edu.cn (M.P.); zhoujiexin@mail.nankai.edu.cn (J.Z.); wangyingying@mail.nankai.edu.cn (Y.W.); zihuawang@mail.nankai.edu.cn (Z.W.); jyotika0201@gmail.com (J.J.); 2 Department of Chemistry, School of Sciences, Hebei University of Science and Technology, Shijiazhuang 050018, China

**Keywords:** graphene, β-lactoglobulin, PtAuPd alloy, electrochemical sensing, glucose sensor

## Abstract

The use of graphene has leapt forward the materials field and the functional modification of graphene has not stopped. In this work, β-lactoglobulin (BLG) was used to functionalize reduced graphene oxide (RGO) based on its amphiphilic properties. Also, trimetallic PtAuPd nanoparticles were reduced to the surface of BLG-functionalized RGO and formed BLG-PtAuPd-RGO nanocomposite using facile synthesis. Transmission electron microscopy, energy-dispersive X-ray spectroscopy and Fourier transform infrared spectra were used to characterize the nanocomposite. Electrocatalytic analysis was evaluated through cyclic voltammetry and chronoamperometry methods. We developed a glucose sensor by fabricating GOD-BLG-PtAuPd-RGO/glassy carbon (GC) electrode. It presented a remarkable sensitivity of 63.29 μA mM^−1^ cm^−2^ (4.43 μA mM^−1^), a wider linear range from 0.005 to 9 mM and a lower detection limit of 0.13 μM (S/N = 3). Additionally, the glucose sensor exhibited excellent testing capability in human serum samples.

## 1. Introduction

Graphene was discovered by Geim et al. in 2004 [1] and it has become a research focus in recent years. It is a carbon allotrope that contains a two-dimensional monoatomic thick building block. Because of its fascinating electronic and mechanical properties, it has been used for research in electrochemical studies. Though graphene is considered to be one of the thinnest and strongest materials [2], it is easy to fold, difficult to separate and remains flat [3,4]. However, combined with other materials, graphene overcomes these problems [5]. Based on the nanocomposites synergies effect, different metal nanomaterials, like Ag [6], Pt [7], Pd [8] and so forth, are being decorated on the graphene nanolamellar surface, forming graphene-metal nanocomposites.

Au, Pt and Pd are currently integrated into nanomaterials to be used in electrochemistry, such as electroanalysis and constructing electrochemical sensors [9,10], due to their extraordinarily physical and chemical properties [11,12,13]. Functions depend on the unique properties. Decorating metal nanomaterials on graphene nanosheets can enhance both the electrocatalytic activity and the electrical conductivity. In addition, because of its nano effect, metal nanomaterials can catalyze specific substances [14].

However, synthesis of a single metal cannot satisfy the performance demands. Therefore, scientists have committed to researching the use of different alloys. Au-Ag alloy nanoparticles with a homogeneous size distribution and good stability were synthesized by Li et al. through simultaneous one-step reduction [15], which is an effective method for detecting many compounds. Research found that Pt-Ni nanorods have higher stability against loss of electrochemically active surface area during potential cycling than Pt nanorods and conventional high-surface-area-carbon-supported Pt nanoparticles [16]. Han et al. reported a relationship between surface composition and catalyst performance in the synthesis of hydrogen peroxide and results indicated that the activity and selectivity could be significantly enhanced using Pd-Au alloy [17]. Also, PtAuCo alloy was synthesized as electrocatalysts that demonstrated enhanced activity and durability toward the Oxygen Reduction Reaction [18]. Additionally, alloys of Pt-Pb [19], Pt-Ni [20], Pb-Au [21] and Pt-Au [22] were synthesized for application in different fields as a new kind of material. These materials indicated that bimetallic nanoparticles exhibit superior properties in comparison with their monometallic counterparts. Therefore, we were inspired to improve the properties of materials through alloys.

The good dispersion of nanocomposites determines their properties of conductivity, catalytic activity and specific surface area. Over the years, researchers have generally used synthetic methods and dispersants to improve the dispersion of graphene-based composite materials. Studies have been done to increase the flexibility of GO through cross-linking with diamine monomers and GO materials pillared with TKAm molecules presented high surface area, precisely defined distance between GO layers and sub-nanometer size of slit pores [23,24]. It showed that by using reactivity between boronic acids and hydroxy groups, GO layers can be linked together to form a new layered structure [25]. Common dispersants include poly dimethyl diallyl ammonium chloride and polyvinyl pyrrolidone non-covalently bonded to graphene [26,27]. In order to improve the biocompatibility and the ability of composite materials to modify bioactive substances more effectively, β-lactoglobulin (BLG) was used for a new function as a dispersant in electrochemical sensing for the first time by our group. Because of its amphiphilic properties, hydrophilic groups adsorbed on the hydrophobic side on graphene surface exposure can facilitate material dispersion. Besides, the structural surface of BLG is rich in sulfhydryl groups that could promote the adsorption of metal nanoparticles [28,29,30]. The uniform distribution of nanoparticles on the surface of graphene also effectively prevents the accumulation of composites.

Diabetes is a disease caused by the inadequate production of insulin by the pancreas, or the inability to effectively utilize insulin [31]. The effective management of diabetes has been intensively studied. As a chronic disease, the development of intelligent monitoring is essential. The application of electrochemical sensing has been widely researches as an effective solution [32,33,34,35]. Many new kinds of testing technology for glucose are appearing constantly. Although scientists have attempted to optimize the detection of glucose, much work remains to be completed on the performance of glucose detection, including using more sophisticated equipment, simplifying sample preparation processes, reducing cost and shortening the testing process and time. Compared with other techniques, such as spectrophotometry [36], ion chromatography [37], chemiluminescence [38], fluorescence [39] and high performance liquid chromatography (HPLC) mass spectrometry [40], electrochemical sensing provides advantages in terms of high selectivity and sensitivity, low cost, rapid response and direct measurement.

In this study, we continued with previous research [41]. The amphiphilic properties of BLG, were used as a dispersant by binding to reduced graphene oxide (RGO). With the help of BLG, nanocomposites could be well distributed. After that, the trimetallic PtAuPd nanoparticles were reduced to the surface of BLG-functionalized RGO, forming BLG-PtAuPd-RGO nanocomposite. Cyclic voltammetry and chronoamperometry were used to study the electrochemical performance and properties in glucose detection. Finally, an amperometric glucose sensor was fabricated by immobilizing glucose oxidase, which was then modified on glassy carbon electrode (GCE). The result showed high sensitivity, low detection limit, fast response and wider linear range compared to other methods. Additionally, a new application of BLG is proposed and a simple synthesis method of trimetallic PtAuPd nanoparticles is introduced. These findings contribute to the study of electrochemical sensing.

## 2. Materials and Methods

### 2.1. Chemicals and Reagents

Graphene oxide (GO) was purchased from Nanjing XFNANO Materials Tech Co. (Nanjing, China). Gold chloride hydrate (HAuCl_4_·3H_2_O, ACS reagent, ≥49.0% Au basis), potassium hexachloroplatinate (≥99.99% trace metals basis), sodium tetrachloropalladate (≥99.99% trace metals basis), ascorbic acid (light sensitive 20–200 MESH Cell Culture Tested), sodium borohydride solution, β-lactoglobulin (from bovine milk ≥90% PAGE), uric acid (≥90% crystalline), bovine serum albumin and glucose oxidase (from Aspergillus niger, catalase ≤0.1 units/mg protein) were obtained from Sigma Aldrich Co (Saint Louis, MO, USA). Glucose, glutaraldehyde and cholestera were obtained from Aladdin Industrial Corporation (Shanghai, China). In this work, all other chemicals were of analytical reagent grade and all relevant experiments used Millipore milli-Q ultrapure water.

### 2.2. Apparatus and Measurements

Transmission electron microscopy (TEM) image analysis was performed on Tecnai G2 F20 instrument (Philips, Amsterdam, The Netherland). Energy-dispersive X-ray (EDX) analysis was carried out on an energy- analyzer equipped on the Tecnai G2 F20 instrument. Fourier transform infrared (FT-IR) spectra was collected on a TENSOR 37 FT-IR (BRUKER, Karlsruhe, Germany). Glucose in real samples from local hospital were calculated by ACCU-CHEK^@^ Performa Connect from Roche Diabetes Care GmbH (South San Francisco, CA, USA).

Electrochemical experiments were carried out on a 283 potentiostat-galvanostat electrochemical workstation (EG&G PARC with M270 software, Microsoft Windows XP) with a conventional three-electrode system, including the modified Pt electrode as the working electrode, a platinum wire (1 mm diameter) as the counter electrode and an Ag/AgCl electrode (saturated with KCl) as the reference electrode.

### 2.3. Electrode Pre-Treatment

Prior to modification, the glassy carbon electrode was polished with 0.3 and 0.05 μm α-alumina powder sequentially. The glassy carbon electrode was placed in 0.5 M H_2_SO_4_, under cyclic voltammograms scanning with 0.3–1.5 V voltage, until the scanning state was stable and then ultrasonically cleaned in doubly distilled water and ethanol for 5 min, respectively. N_2_ was used to blow dry electrodes.

### 2.4. Preparation of the Modified Sensing Electrodes

**Scheme 1:**
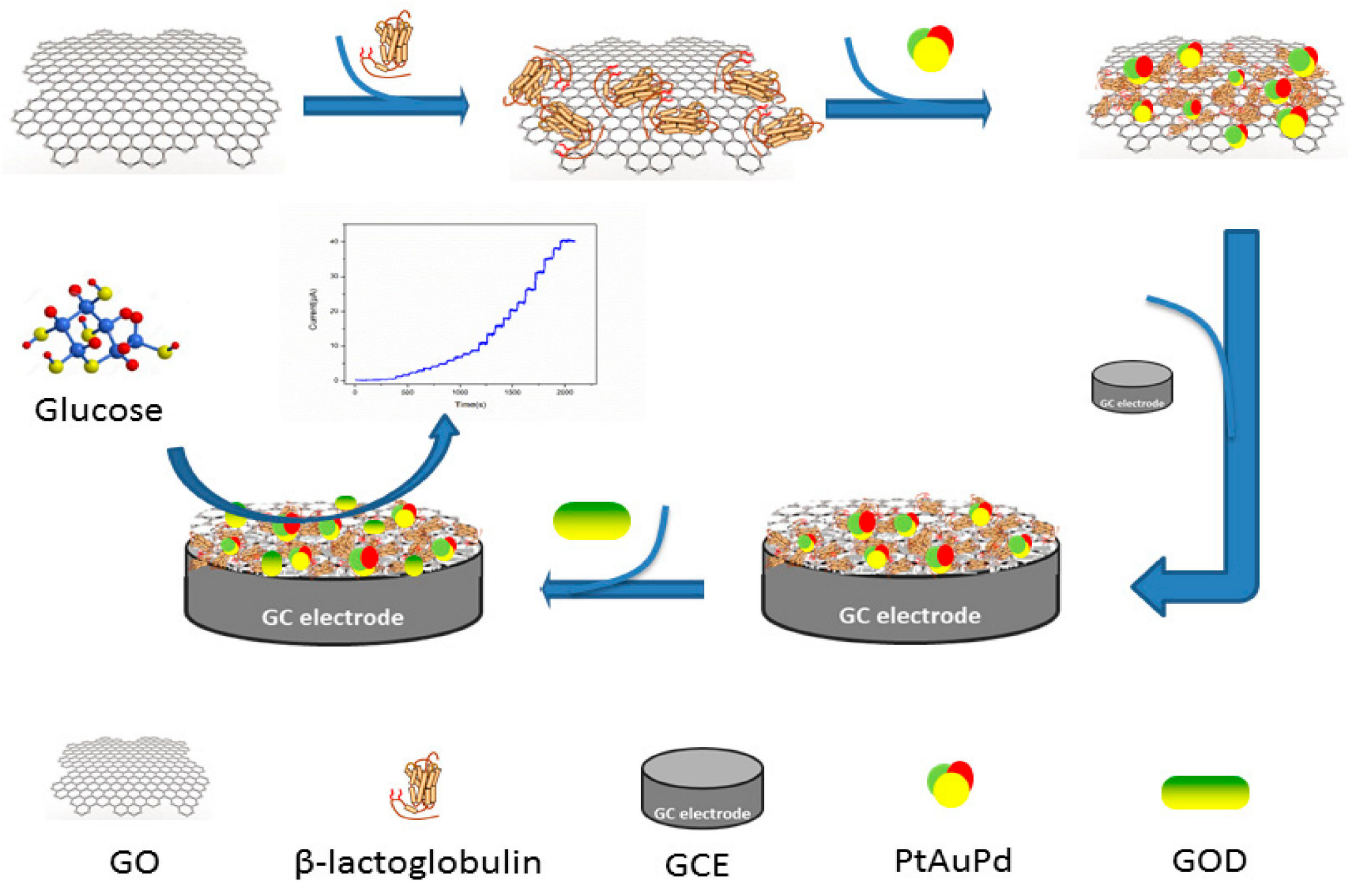
The preparation of glucose sensor.

In this study, 20 mg of graphene oxide (GO) were uniformly dispersed in 20 mL of doubly distilled water with sonication for 1 h. Next, 1 mL of 3 mg mL^−1^ BLG was added and was stirred until temperature decreased to 25 °C. Then, 60 µL of 1 M gold chloride hydrate, 60 µL of 1 M potassium hexachloroplatinate and 60 µL of 1 M sodium tetrachloropalladate, were added dropwise into the suspension, mixing fully one by one, under sustained stirring at indoor temperature. Then slowly, the configured 5 mL sodium borohydride solution was added dropwise until the color changed gradually into deep black. Finally, BLG-PtAuPd-RGO nanocomposite was obtained by centrifugation, washed repeatedly 5 times and spare kept at room temperature. Then, 5 mg of prepared BLG-PtAuPd-RGO composite materials was dissolved in 5 mL deionized water and ultrasonicated for 1 h to make it well dispersed. GOD from the ultra-low temperature freezer was configured to 2 mg/mL of glucose oxidase solution mixed with glutaraldehyde and bovine serum albumin. Then, 5 μL of BLG-PtAuPd-RGO suspension solution was immobilized on the surface of the GC electrode and 5 μL of GOD was further immobilized on the BLG-PtAuPd-RGO-modified GC electrode surface to fabricate a glucose sensor, denoted as GOD-BLG-PtAuPd-RGO/GCE, which was stored at 4 °C. The preparation is shown in Scheme 1.

## 3. Results and Discussion

### 3.1. Characterization of BLG-PtAuPd-RGO

In the synthesis and application of nanomaterials, the dispersion effect and stability of nanomaterials directly influence their application in different fields as functional materials. At the same time, many efforts have been made to optimize the dispersion effect of composite materials. The orderliness of composite materials improves its conductivity, catalytic activity and other properties [26,27]. In order to improve the biocompatibility and the ability of composite materials to more effectively modify bioactive substances, β-lactoglobulin (BLG) was used for a new function as a dispersant in electrochemical sensing for the first time by our group.

**Figure 1:**
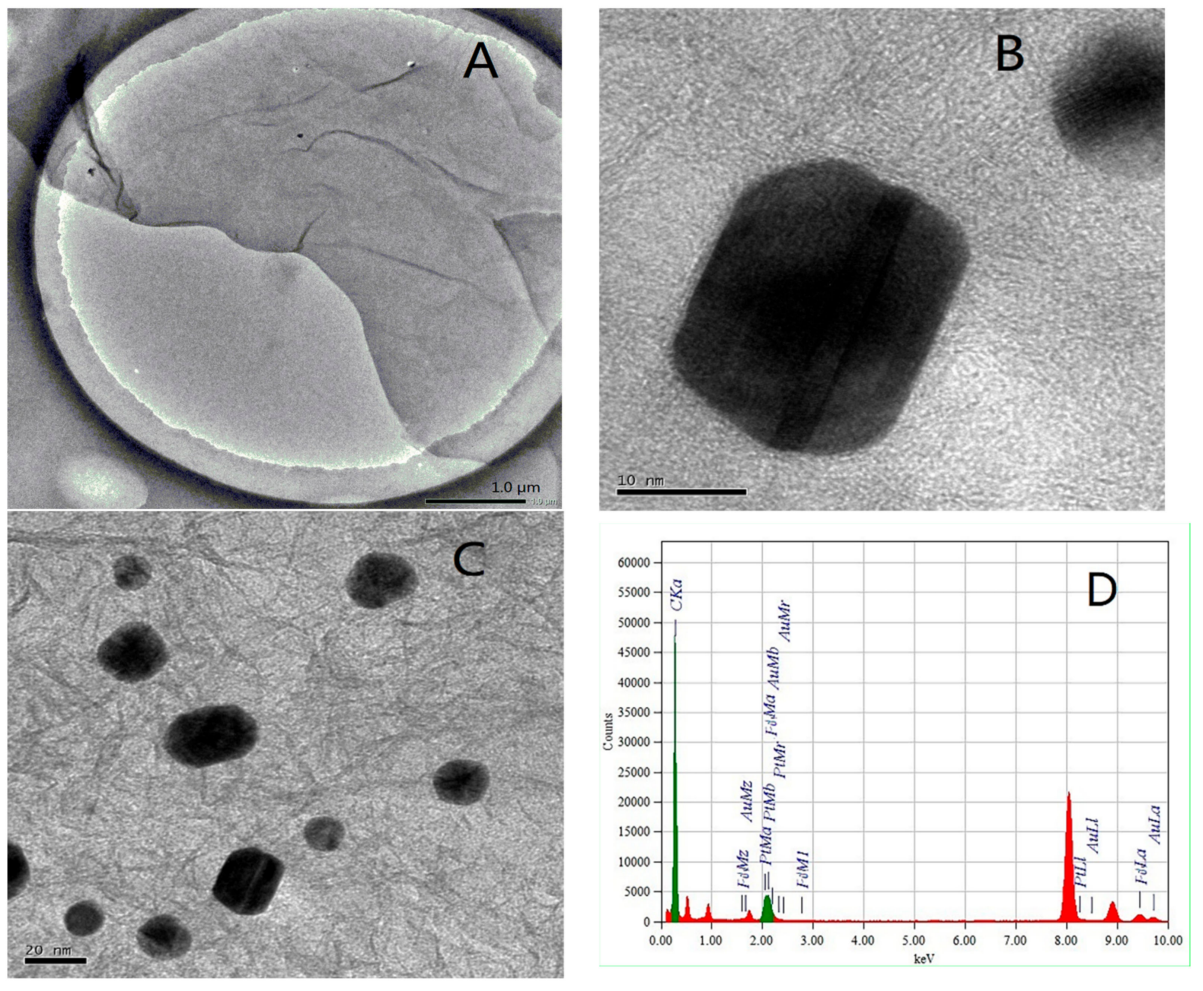
Transmission electron microscopy (TEM) images of (**A**) graphene oxide (GO) with scale bar of 1.0 μm, (**B**,**C**) BLG-PtAuPd-RGO with scale bar of 10 nm and 20 nm; electron-dispersive X-ray (EDX) spectrum (**D**) of BLG-PtAuPd-RGO nanocomposite.

In this part, the morphologies of the GO and BLG-PtAuPd-RGO were characterized by TEM and as shown in Figure 1. As can be seen from the Transmission electron microscope results in Figure 1A, graphene oxide had an obvious laminar structure. However, functional modification of graphene decreases the oxygen-containing functional groups on the surface of graphene, causing more folds and accumulation. The combination of BLG could effectively change the accumulation of composite materials and the introduction of PtAuPd alloys could disperse more evenly on the surface of composite materials, as shown in Figure 1B,C. At the same time, we also observed that the diameter of the PtAuPd alloy was about 20 nm and so we performed EDX analysis to further study the composition of elements from BLG-PtAuPd-RGO (Figure 1D). The results showed that the absorption peak of elemental composition was obvious; the absorption peaks of palladium, gold and platinum elements were due to the PtAuPd alloys; and carbon absorption peak was attributed to the RGO. Figure S1 shows the result of FTIR. We could see that in curve a (GO) the special absorption peak position of 3429 cm^−1^, 1729 cm^−1^, 1625 cm^−1^, 1219 cm^−1^ and 1055 cm^−1^ corresponding absorption peak chemical bonds of O–H, C=O, C=C, C–O–C and C–O in GO. In curve b (BLG-PtAuPd-RGO) the absorption peak in 3429 cm^−1^ (O–H), 1219 cm^−1^ (C–O–C), 1055 cm^−1^ (C–O) decreased and smoothed, which means that GO was reduced to RGO. At the same time the absorption peak at 1729 cm^−1^ (C=O) decreased because trimetallic PtAuPd was attached to graphene materials. The dispersion performance of BLG-PtAuPd-RGO is more directly demonstrated in Figure S2. We left composite materials with and without BLG in a static position at room temperature for 48 h and observed that BLG-PtAuPd-RGO had excellent dispersion effect. In conclusion, BLG-PtAuPd-RGO nanocomposites were successfully synthesized.

### 3.2. Electrochemical Performance of BLG-PtAuPd-RGO/GCE

**Figure 2:**
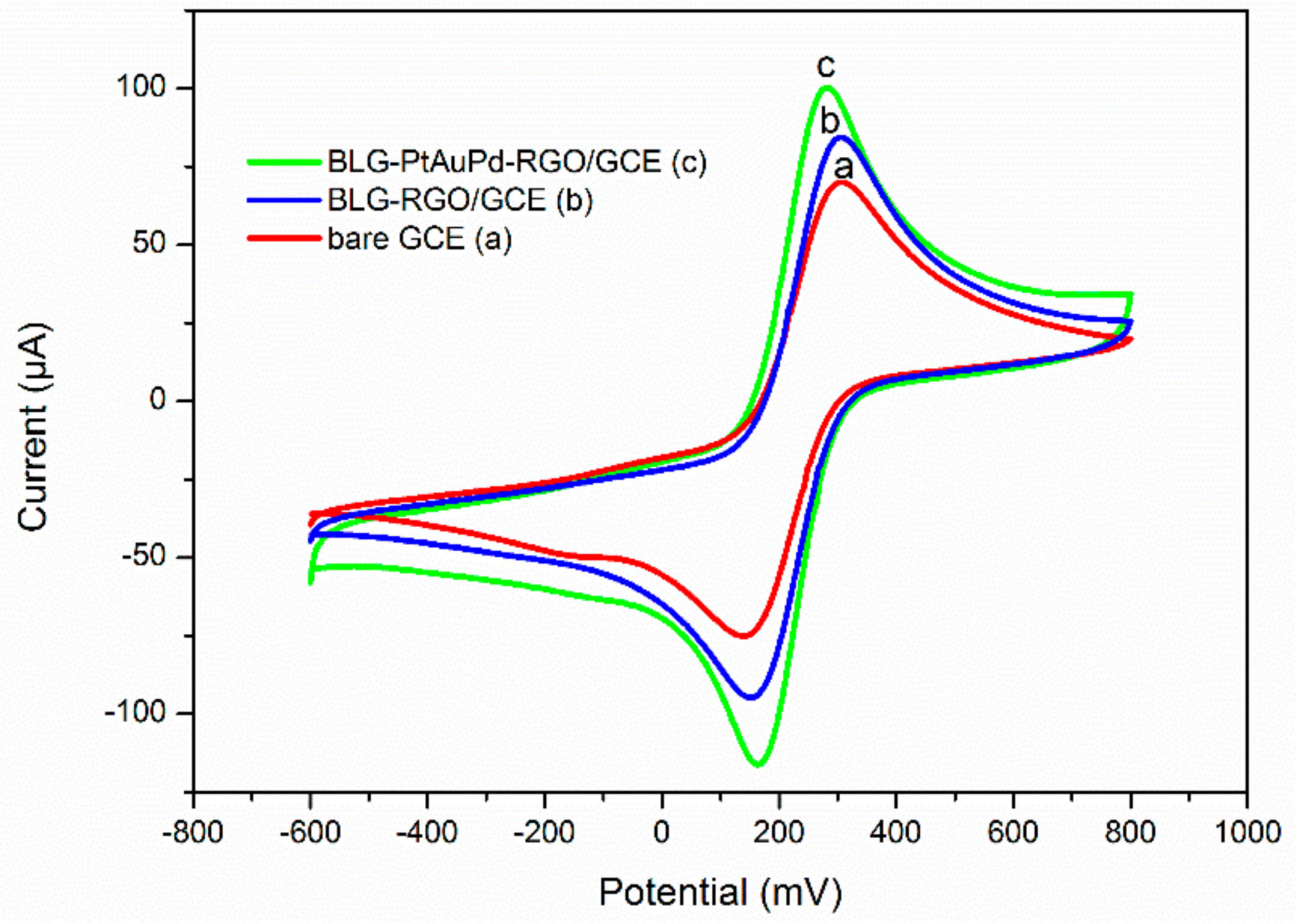
CVs of (**a**) bare glassy carbon (GC) electrode; (**b**) BLG-RGO/GC electrode; (**c**) BLG-PtAuPd-RGO/GC electrode recorded in 0.1 M KCl solution containing 10 mM [Fe(CN)_6_]^3−^.

Based on previous composite materials, BLG-RGO and BLG-PtAuPd-RGO were synthesized under the same conditions to modify the GC electrode. Different modified electrodes were used for electrochemical analysis using the cyclic voltammetry method. As shown in Figure 2, compared with a bare GC electrode and other modified electrodes, it was obvious that BLG-PtAuPd-RGO/GCE presented an excellent electrochemical performance under conditions of 0.1 M KCl aqueous containing 10 mM K_3_[Fe(CN)^6^] (pH 7.0, scan rate: 50 mV s^−1^, scan area from −600 to 800 mV). The peak of the cyclic voltammetry curve with different modified electrodes reflected the oxidation reduction of Fe^2+/3+^. Contrasting bare GCE (a), BLG-RGO/GCE (b) and BLG-PtAuPd-RGO/GCE (c) showed peaks increased. Also, BLG-PtAuPd-RGO/GCE (c) increased dramatically. Besides, by calculating dependence on the Randles-Sevcik equation as follows [42], we obtained the effective areas of different modified electrodes.

*I_p_* = 2.69 × 10^5^*AD*^1/2^*n*^3/2^*v*^1/2^*C* (1)

where *I_p_* relates to the redox peak current; *A* represents the electrode’s electroactive surface area (cm^2^); *D* means the diffusion coefficient of the molecule in solution, which was (6.70 ± 0.02) × 10^−6^ cm^2^ s^−1^; *n* is the number of electron participating in the reaction which was equal to 1; *v* is the scan rate (V s^−1^); and *C* corresponds to the concentration of the probe molecule in the solution, which was 10 mM. According to the above equation, the electroactive surface area of BLG-PtAuPd-RGO/GCE was calculated to be 1.2 and 1.5 times higher than BLG-RGO/GCE and bare GC electrode, respectively.

### 3.3. Electrocatalytic Activity of GOD-BLG-PtAuPd-RGO/GCE

**Figure 3:**
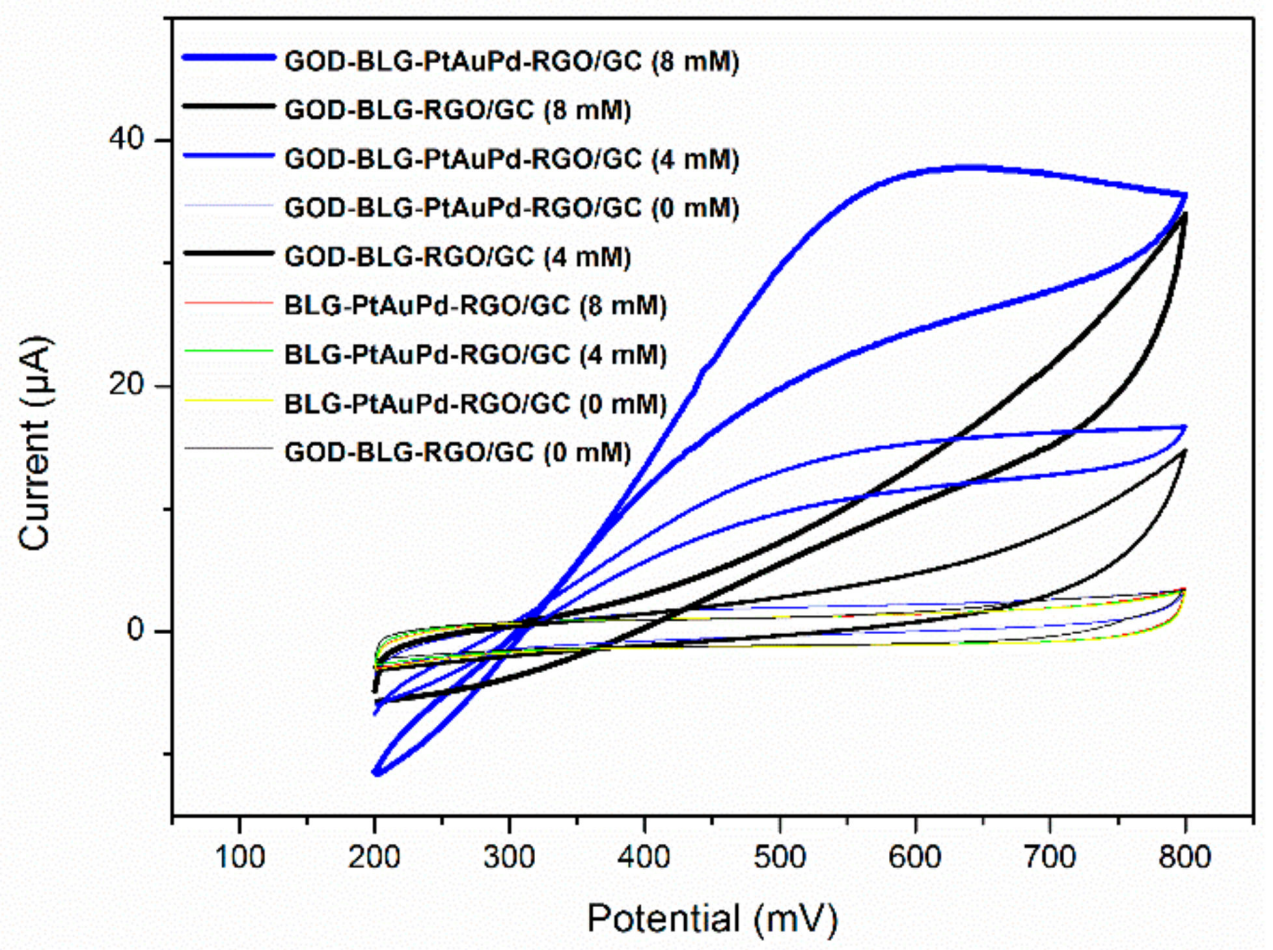
CVs response of GOD-BLG-PtAuPd-RGO/GC, GOD-BLG-RGO/GC and BLG-PtAuPd-RGO/GC electrodes upon different concentration additions with 0 mM, 4 mM and 8 mM glucose into pH 7.0, 0.1 M phosphate buffered solution (PBS) under stirring.

Cyclic voltammetry was used to study the electrocatalytic activity of GOD-BLG-PtAuPd-RGO/GCE towards detection with and without glucose. In Figure 3, there was not any current responses when detected without glucose. The BLG-PtAuPd-RGO/GCE which without GOD presented no current responses with and without glucose. The control group showed current responses when 4 mM and 8 mM glucose was added, indicated that both GOD-BLG-RGO/GCE and GOD-BLG-PtAuPd-RGO/GCE could catalyze and detect glucose and the current response of the oxidation characteristic peak increased with increasing glucose concentration. The anodic catalytic current generated due to the oxidation of hydrogen peroxide, a catalyzed product from GOD. However, compared to GOD-BLG-RGO/GCE, GOD-BLG-PtAuPd-RGO/GCE showed better catalytic effect and lower working potential of oxidation characteristic peak due to the help of PtAuPd alloy. Therefore, GOD-BLG-PtAuPd-RGO/GCE was the optimal sensor interface of glucose sensor. Also, when glucose was added in the solution of 0.1 M phosphate buffered solution (PBS) pH 7.0 at potential from 200 to 800 mV with GOD-BLG-PtAuPd-RGO/GCE, there was an obvious current response peak around 600 mV. This provided the working voltage for our research to further study the GOD-BLG-PtAuPd-RGO/GCE sensing interface using chronometric current method.

**Figure 4:**
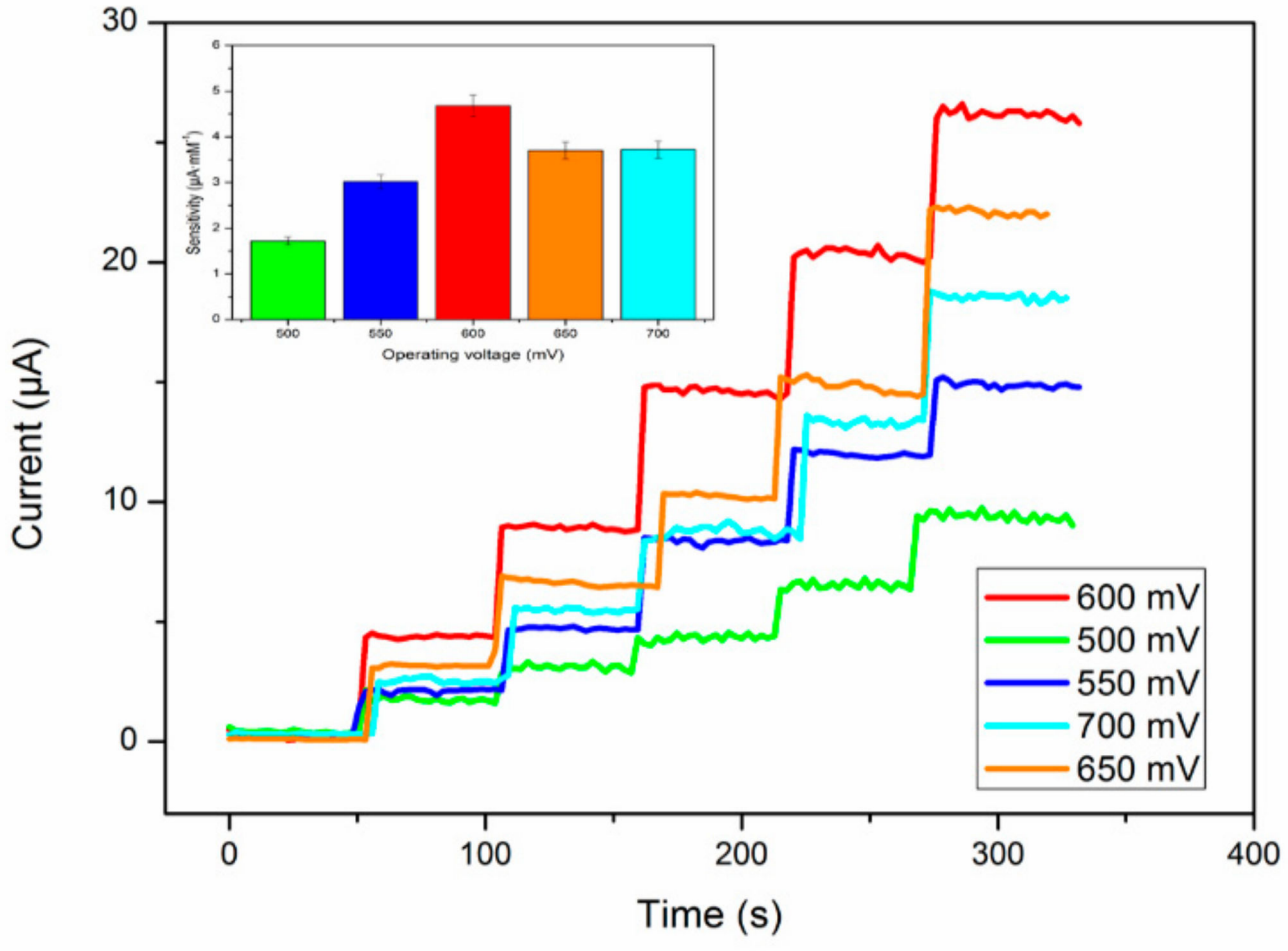
Amperometric response of GOD-BLG-PtAuPd-RGO/GC electrode upon successive additions of 1 mM glucose into 0.1 M PBS solution under stirring at operating voltage from 500 mV to 700 mV.

In order to further the study of working voltage, we performed experiments on the amperometric responses of GOD-BLG-PtAuPd-RGO/GCE under a series of operating voltages with successive addition of 1 mM glucose through chronoamperometry. The optimization results of working voltage are shown in Figure 4. After setting different working voltages at 500, 550, 600, 650 and 700 mV with GOD-BLG-PtAuPd-RGO/GCE in 0.1 M PBS pH 7.0, we obtained the amperometric responses by successive addition of 1 mM glucose (Figure 4). The inset figure shows sensitivity of the ampere response under different optimization conditions. According to this, we concluded that the optimal working voltage for glucose detection with GOD-BLG-PtAuPd-RGO/GCE is 600 mV. Therefore, we chose the optimum working voltage to complete the following research.

### 3.4. Chronoamperometric Responses of GOD-BLG-PtAuPd-RGO/GCE

**Figure 5:**
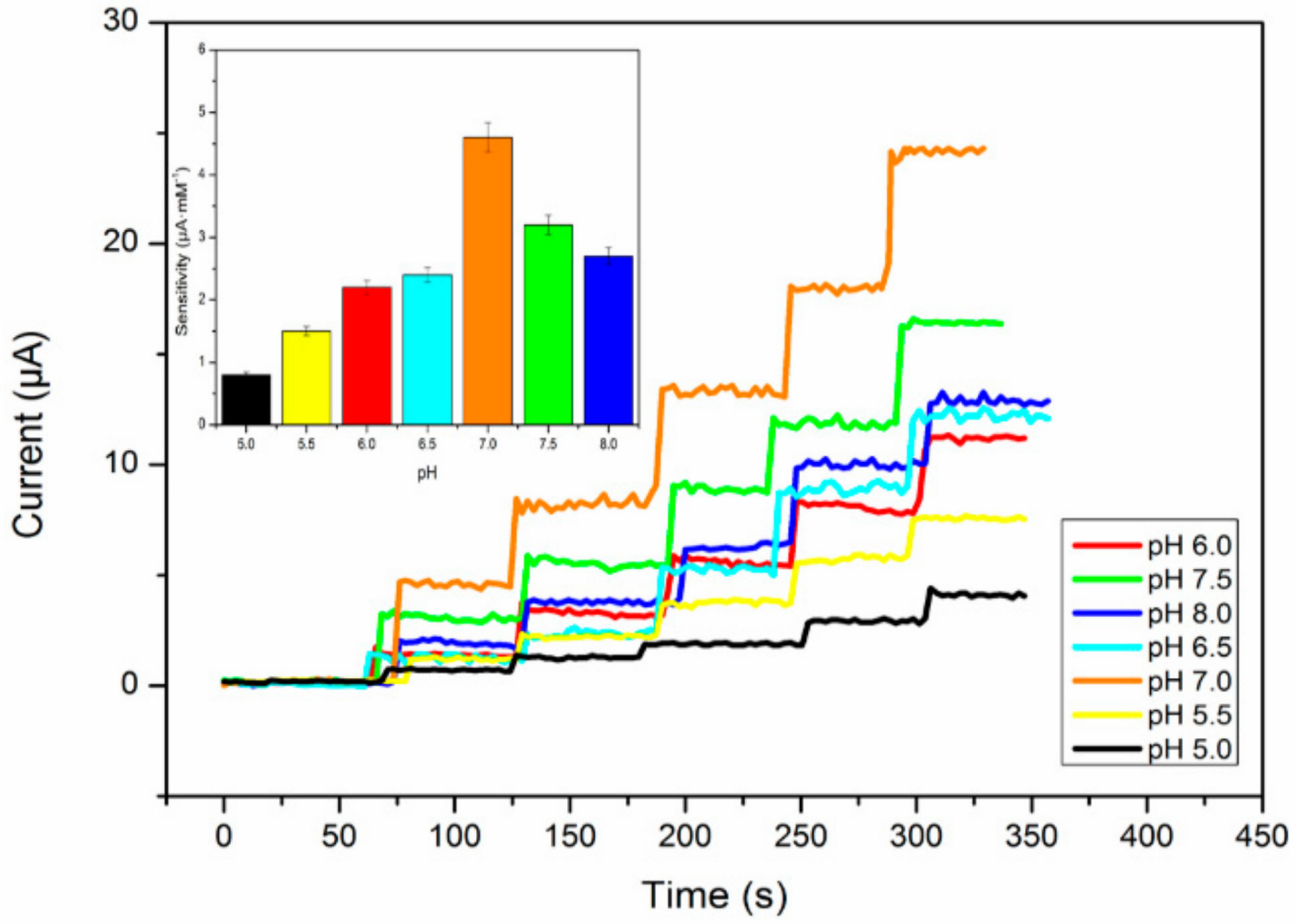
Amperometric response of GOD-BLG-PtAuPd-RGO/GC electrode upon successive additions of 1 mM glucose into 0.1 M PBS solution under stirring at operating voltage of 600 mV with different pH values from 5.0 to 8.0.

The pH of the testing environment had a significant impact on the detection results of glucose, so we optimized the pH value of the detection system. The result is shown in Figure 5, with the pH values of 5.0, 5.5, 6.0, 6.5, 7.0, 7.5 and 8.0, different amperometric responses are presented. Compared with other reports, pH 7.0 provided best sensitivity (inset in Figure 5), so this was selected as the optimum pH for experimental condition.

**Figure 6:**
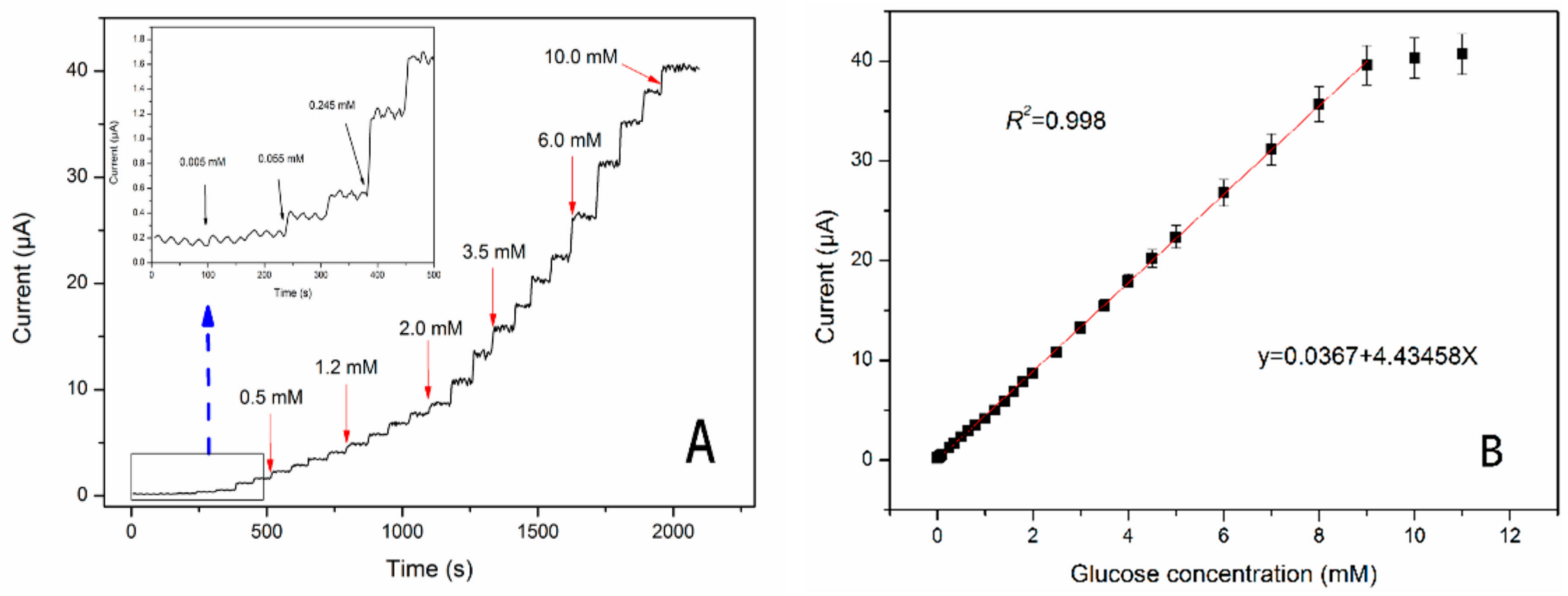
(**A**) Amperometric response of GOD-BLG-PtAuPd-RGO/GC electrode upon successive additions of glucose from 0.005 to 9 mM into 0.1 M pH 7.0 PBS solution under stirring at operating voltage of 600 mV; (**B**) The calibration curve between amperometric response and glucose concentration.

Attributed to the BLG-functionalized RGO and trimetallic PtAuPd nanocomposite, BLG-PtAuPd-RGO provided excellent electron-transfer kinetics, larger active surface area and superior electrocatalytic activity. These characteristics allowed have the advantages of a larger linear range, greater sensitivity and lower detection limit after GOD was immobilized on the BLG-PtAuPd-RGO-modified GC electrode surface to fabricate a glucose sensor. As shown in Figure 6A, as the glucose concentration increased, glucose concentration was positively correlated with the current responses. The increase in current also presented a dynamic change process that was rapid at the initial stage and then gradually increased with the further increase in glucose concentration. We concluded that the time of current response reached 95% of steady-state current within five seconds using GOD-BLG-PtAuPd-RGO/GCE fabricated glucose sensor.

Besides, a calibration curve was created as presented in Figure 6B. We discovered that the fabricated glucose sensor showed a wider linear range from 0.005 to 9 mM by performing five repetitive experiments. The sensitivity was calculated to be 63.29 μA mM^−^^1^ cm^−2^ (4.43 μA mM^−1^) with a lower detection limit of 0.13 μM (*n* = 5, *R*^2^ = 0.998, S/N = 3). The resulting properties are completely suitable for the diagnosis of diabetes. Compared with other glucose electrochemical detection studies, especially the previous research by our group on BLG, listed in Table 1, the performance our method was much improved.

Furthermore, to research the selectivity of the prepared GOD-BLG-PtAuPd-RGO/GCE fabricated glucose sensor, we designed an experiment using the chronoamperometry method. There was a current response after adding 0.5 mM glucose, then other interfering substances such as 0.6 mM cholestera, 0.8 mM uric acid and 0.6 mM ascorbic acid were added and found no significant current responses, However, when adding 0.5 mM glucose again it showed current response, thus illustrating that the GOD-BLG-PtAuPd-RGO/GCE fabricated glucose sensor had good selectivity (Figure S3). In order to test the application of the glucose sensor in real samples, five human serums from local hospital were analyzed. The glucose concentration in real samples were calculated by ACCU-CHEK Performa devices in local hospital. Conditions were set to serum levels with multiple glucose standard and result could be found in Table 2.

### 3.5. Stability and Reproducibility of the GOD-BLG-PtAuPd-RGO/GCE Fabricated Glucose Biosensor

**Figure 7:**
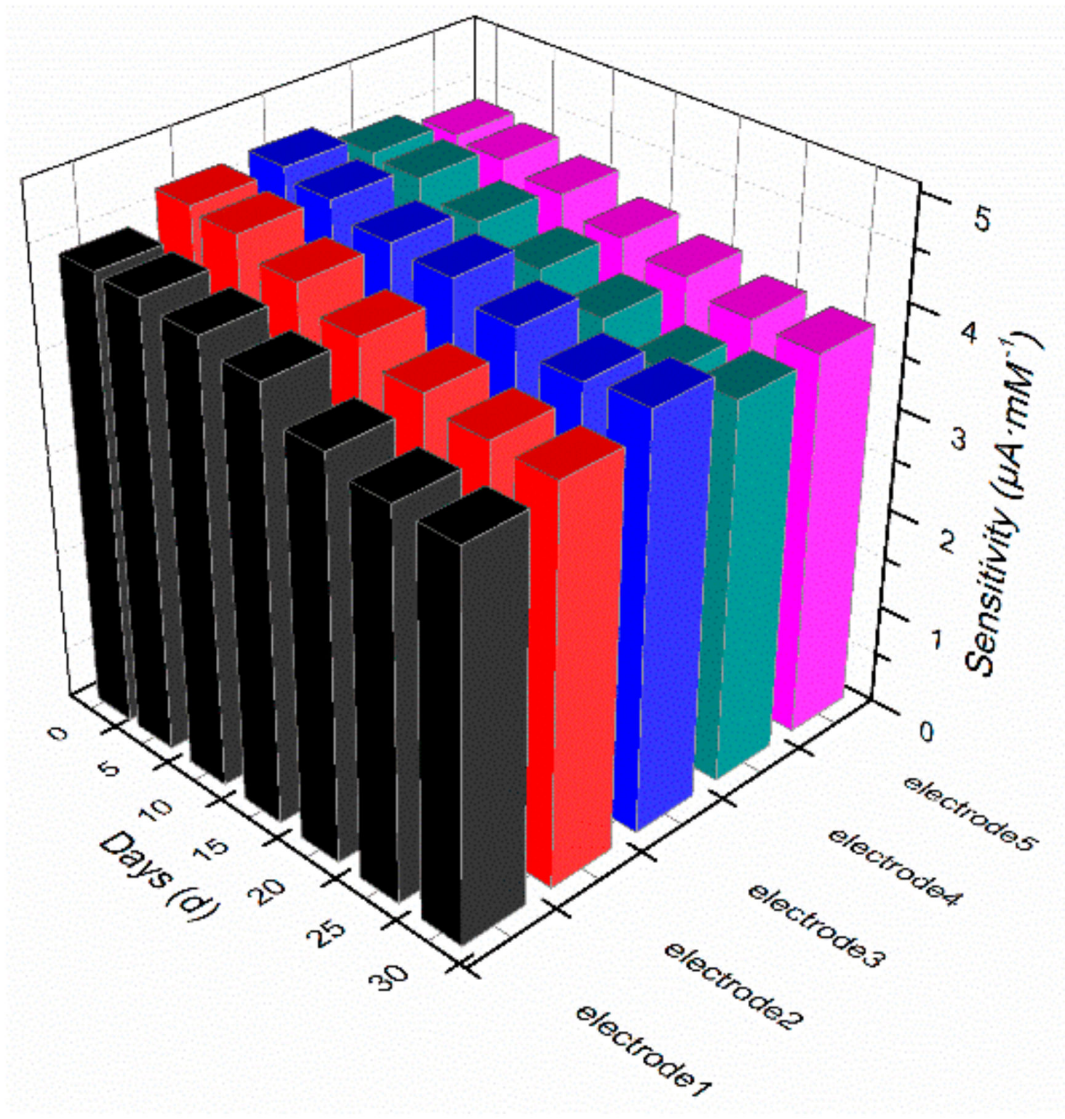
Research of stability and reproducibility of five GOD-BLG-PtAuPd-RGO/GC electrodes upon successive additions of glucose detecting every five days and continued for 30 days.

We performed a series of studies on stability using the same GOD-BLG-PtAuPd-RGO/GCE for glucose detection every five days and continued for a month. GOD-BLG-PtAuPd-RGO/GCE was stored in freezer at 4 °C when not used. The electrode maintained an initial current response of 84.7% over a month. Also, five GOD-BLG-PtAuPd-RGO/GC electrodes were prepared at the same conditions for research of reproducibility. The relative standard deviation of the sensitivities was calculated to be 3.15%. The result indicated that GOD-BLG-PtAuPd-RGO/GCE fabricated glucose sensor had excellent stability and reproducibility toward glucose (Figure 7).

## 4. Conclusions

In this study, a facile synthesis of β-lactoglobulin-functionalized reduced graphene oxide and trimetallic PtAuPd nanocomposite was synthesized for the first time and applied to electrochemical sensing for the detection of glucose. Based on the amphiphilic properties of BLG, the nanocomposite was used as a dispersant by binding to RGO. With the help of trimetallic PtAuPd nanoparticles, the electrochemical properties of nanocomposites were considerably improved. The glucose sensor showed excellent application in real samples and would contribute to the diagnoses and treatment of diabetes. Additionally, as a functional protein dispersant, we think that it would have broad application prospects in scientific research.

## Figures and Tables

**Table 1:** Characteristics of various reported glucose biosensors.

Electrode Composite Materials	Linearity (mM)	Detection Limit (μM)	Sensitivity (μA mM^−1^ cm^−2^)	pH	Reference
GOD−Ag@C/Nafion/GCE	0.05–2.5	20	24.65	7.0	[43]
GOx-AuNPs-PVP ^a^-PANI ^b^/GCE	0.05–2.25	10	9.62	7.0	[44]
4ATP ^c^/PVA ^d^/PEI ^e^/AuNPs/GOx/AuE	0.01–0.2	0.9	-	5.0	[32]
GOx/TCS−TiO_2_^f^/chitosan/GCE	0.005–1.32	2.0	23.2	7.0	[45]
bMWCNTs-HBPEI ^g^/AuNPs-B(OH)_2_/GOx/GCE	0.25–5	0.8	28.6	7.4	[35]
GOD/BLG/multi wall carbon nanotubes/gold nanoparticles/GCE	0.025–5.5	1.1	56.3	7.0	[41]
GOD-BLG-PtAuPd-RGO/GCE	0.005–9	0.13	63.29	7.0	This work

^a^ Polyvinylpyrrolidone. ^b^ Polyaniline. ^c^ 4-aminothiophenol. ^d^ Poly(vinyl alcohol). ^e^ Poly(ethyleneimine). ^f^ Tetragonal columnar-shaped TiO_2_. ^g^ Hyperbranched polyethyleneimine.

**Table 2:** Determination of glucose in real human serum sample with fabricated sensor.

Sample	Calculated by Local Hospital (mM)	Detected by Fabricated Sensor (mM)	Glucose Added (mM)	Glucose Found (mM)	Recovery (%)	RSD (%, *n* = 5)
1	4.20	4.19	0.2	4.38	95.00	2.79
2	3.62	3.64	0.4	4.03	97.50	3.37
3	3.91	3.92	0.6	4.53	101.67	2.45
4	4.17	4.20	0.8	5.12	102.50	2.89
5	4.58	4.56	1.0	5.52	96.00	3.05

## Supplementary Materials

The following are available online at http://www.mdpi.com/2079-4991/8/9/724/s1, Figure S1: FT-IR patterns of GO (a) and BLG-PtAuPd-RGO (b), Figure S2: Synthetic composite materials with BLG (B) and without BLG (A) in a static position at indoor temperature for 48 hours, Figure S3: Amperometric response of GOD-BLG-PtAuPd-RGO/GC electrodes in 0.1 M PBS (PH 7.0) solution mixture at operating voltage of 600 mV with 0.5 mM glucose or other substance: uric acid (0.8 mM), ascorbic acid (0.6 mM) and cholesterol (0.6 mM).

## References

[1] Novoselov K.S., Geim A.K., Morozov S.V., Jiang D., Zhang Y., Dubonos S.V., Grigorieva I.V., Firsov A.A. (2004). Electric field effect in atomically thin carbon films. Science.

[2] Dai G., Mishnaevsky L. (2014). Graphene reinforced nanocomposites: 3D simulation of damage and fracture. Comput. Mater. Sci..

[3] He H., Gao C. (2010). General approach to individually dispersed, highly soluble, and conductive graphene nanosheets functionalized by nitrene chemistry. Chem. Mater..

[4] Goh M.S., Pumera M. (2010). Single-, few-, and multilayer graphene not exhibiting significant advantages over graphite microparticles in electroanalysis. Anal. Chem..

[5] Zhu J., Zhu T., Zhou X., Zhang Y., Lou X.W., Chen X., Zhang H., Hng H.H., Yan Q. (2011). Facile synthesis of metal oxide/reduced graphene oxide hybrids with high lithium storage capacity and stable cyclability. Nanoscale.

[6] Zhang D., Liu X., Wang X. (2011). Green synthesis of graphene oxide sheets decorated by silver nanoprisms and their anti-bacterial properties. J. Inorg. Biochem..

[7] Yoo E., Okata T., Akita T., Kohyama M., Nakamura J., Honma I. (2009). Enhanced electrocatalytic activity of Pt subnanoclusters on graphene nanosheet surface. Nano Lett..

[8] Shankar S.R., Cristina G.N., Kannan B., Marko B., Klaus K. (2008). Electrochemical modification of graphene. Adv. Mater..

[9] Łuczak T. (2009). Comparison of electrochemical oxidation of epinephrine in the presence of interfering ascorbic and uric acids on gold electrodes modified with s-functionalized compounds and gold nanoparticles. Electrochim. Acta.

[10] Luque G.L., Ferreyra N.F., Granero A., Bollo S., Rivas G.A. (2011). Electrooxidation of DNA at glassy carbon electrodes modified with multiwall carbon nanotubes dispersed in polyethylenimine. Electrochim. Acta.

[11] Yue R., Shan L., Yang X., Zhang W. (2012). Approaches to target profiling of natural products. Curr. Med. Chem..

[12] Hirano I., Imaoka T., Yamamoto K. (2014). Deposition of the monodispersed pt nanodots on a substrate by using the pt nanoparticle-containing dendrimer micelle aqueous solution. J. Inorg. Organomet. Polym. Mater..

[13] Liu Y., Feng X., Shen J., Zhu J.-J., Hou W. (2008). Fabrication of a novel glucose biosensor based on a highly electroactive polystyrene/polyaniline/au nanocomposite. J. Phys. Chem. B.

[14] Hu J., Li F., Wang K., Han D., Zhang Q., Yuan J., Niu L. (2012). One-step synthesis of graphene–AuNPs by HMTA and the electrocatalytical application for O_2_ and H_2_O_2_. Talanta.

[15] Li S.-F., Zhang X.-M., Yao Z.-J., Yu R., Huang F., Wei X.-W. (2009). Enhanced chemiluminescence of the rhodamine 6G−Cerium(IV) system by Au−Ag alloy nanoparticles. J. Phys. Chem. C.

[16] Kariuki N.N., Khudhayer W.J., Karabacak T., Myers D.J. (2013). Glad Pt–Ni alloy nanorods for oxygen reduction reaction. ACS Catal..

[17] Han Y.-F., Zhong Z., Ramesh K., Chen F., Chen L., White T., Tay Q., Yaakub S.N., Wang Z. (2007). Au promotional effects on the synthesis of H_2_O_2_ directly from H_2_ and O_2_ on supported Pd−Au alloy catalysts. J. Phys. Chem. C.

[18] Tan X., Prabhudev S., Kohandehghan A., Karpuzov D., Botton G.A., Mitlin D. (2015). Pt–Au–Co alloy electrocatalysts demonstrating enhanced activity and durability toward the oxygen reduction reaction. ACS Catal..

[19] Mercer M.P., Plana D., Fermίn D.J., Morgan D., Vasiljevic N. (2015). Growth of epitaxial Pt_1−x_Pb_x_ alloys by surface limited redox replacement and study of their adsorption properties. Langmuir.

[20] Roy S., Hariharan S., Tiwari A.K. (2018). Pt–Ni subsurface alloy catalysts: An improved performance toward CH_4_ dissociation. J. Phys. Chem. C.

[21] Kim Y.-G., Kim J.Y., Thambidurai C., Stickney J.L. (2007). Pb deposition on I-coated Au(111). UHV-EC and EC-STM studies. Langmuir.

[22] Yang Z., Yang X., Xu Z. (2008). Molecular dynamics simulation of the melting behavior of Pt−Au nanoparticles with core−shell structure. J. Phys. Chem. C.

[23] Sun J., Morales-Lara F., Klechikov A., Talyzin A.V., Baburin I.A., Seifert G., Cardano F., Baldrighi M., Frasconi M., Giordani S. (2017). Porous graphite oxide pillared with tetrapod-shaped molecules. Carbon.

[24] Hung W.-S., Tsou C.-H., De Guzman M., An Q.-F., Liu Y.-L., Zhang Y.-M., Hu C.-C., Lee K.-R., Lai J.-Y. (2014). Cross-linking with diamine monomers to prepare composite graphene oxide-framework membranes with varying d-spacing. Chem. Mater..

[25] Burress J.W., Gadipelli S., Ford J., Simmons J.M., Zhou W., Yildirim T. (2010). Graphene oxide framework materials: Theoretical predictions and experimental results. Angew. Chem. Int. Ed. Engl..

[26] Zhang C., Zhang Y., Miao Z., Ma M., Du X., Lin J., Han B., Takahashi S., Anzai J.I., Chen Q. (2016). Dual-function amperometric sensors based on poly(diallydimethylammoniun chloride)-functionalized reduced graphene oxide/manganese dioxide/gold nanoparticles nanocomposite. Sens. Actuators B Chem..

[27] Zhang Z., Yin L. (2016). Polyvinyl pyrrolidone wrapped Sn nanoparticles/carbon Xerogel composite as anode material for high performance lithium ion batteries. Electrochim. Acta.

[28] Sahihi M., Ghayeb Y., Bordbar A.K. (2013). Interaction of β-lactoglobulin with resveratrol: Molecular docking and molecular dynamics simulation studies. Chem. Biochem. Eng. Q..

[29] Fragneto G., Su T.J., Lu J.R., Thomas R.K., Rennie A.R. (2000). Adsorption of proteins from aqueous solutions on hydrophobic surfaces studied by neutron reflection. Phys. Chem. Chem. Phys..

[30] Feng X., Hu J., Chen X., Xie J., Liu Y. (2009). Synthesis and electron transfer property of sulfhydryl-containing multi-walled carbon nanotube/gold nanoparticle heterojunctions. J. Phys. D Appl. Phys..

[31] Hwang D.-W., Lee S., Seo M., Chung T.D. (2018). Recent advances in electrochemical non-enzymatic glucose sensors—A review. Anal. Chim. Acta.

[32] Sapountzi E., Braiek M., Vocanson F., Chateaux J.-F., Jaffrezic-Renault N., Lagarde F. (2017). Gold nanoparticles assembly on electrospun poly(vinyl alcohol)/poly(ethyleneimine)/glucose oxidase nanofibers for ultrasensitive electrochemical glucose biosensing. Sens. Actuators B Chem..

[33] Uehara H., Kakiage M., Sekiya M., Sakuma D., Yamonobe T., Takano N., Barraud A., Meurville E., Ryser P. (2009). Size-selective diffusion in nanoporous but flexible membranes for glucose sensors. ACS Nano.

[34] Kim I., Kwon D., Lee D., Lee T.H., Lee J.H., Lee G., Yoon D.S. (2018). A highly permselective electrochemical glucose sensor using red blood cell membrane. Biosens. Bioelectron..

[35] Eguílaz M., Villalonga R., Pingarrón J.M., Ferreyra N.F., Rivas G.A. (2015). Functionalization of bamboo-like carbon nanotubes with 3-mercaptophenylboronic acid-modified gold nanoparticles for the development of a hybrid glucose enzyme electrochemical biosensor. Sens. Actuators B Chem..

[36] Rocha D.L., Rocha F.R.P. (2010). A flow-based procedure with solenoid micro-pumps for the spectrophotometric determination of uric acid in urine. Microchem. J..

[37] Zhao F.Y., Wang Z.H., Wang H., Zhao R., Ding M.Y. (2011). Determination of uric acid in human urine by ion chromatography with conductivity detector. Chin. Chem. Lett..

[38] Wu F., Huang Y., Li Q. (2005). Animal tissue-based chemiluminescence sensing of uric acid. Anal. Chim. Acta.

[39] Galbán J., Andreu Y., Almenara M.J., de Marcos S., Castillo J.R. (2001). Direct determination of uric acid in serum by a fluorometric-enzymatic method based on uricase. Talanta.

[40] Perelló J., Sanchis P., Grases F. (2005). Determination of uric acid in urine, saliva and calcium oxalate renal calculi by high-performance liquid chromatography/mass spectrometry. J. Chromatogr. B.

[41] Du X., Miao Z., Zhang D., Fang Y., Ma M., Chen Q. (2014). Facile synthesis of beta-lactoglobulin-functionalized multi-wall carbon nanotubes and gold nanoparticles on glassy carbon electrode for electrochemical sensing. Biosens. Bioelectron..

[42] Ngamchuea K., Eloul S., Tschulik K., Compton R.G. (2014). Planar diffusion to macro disc electrodes—What electrode size is required for the Cottrell and Randles-Sevcik equations to apply quantitatively?. J. Solid State Electrochem..

[43] Zhou X., Dai X., Li J., Long Y., Li W., Tu Y. (2015). A sensitive glucose biosensor based on Ag@C core–shell matrix. Mater. Sci. Eng. C.

[44] Miao Z., Wang P., Zhong A., Yang M., Xu Q., Hao S., Hu X. (2015). Development of a glucose biosensor based on electrodeposited gold nanoparticles-polyvinylpyrrolidone-polyaniline nanocomposites. J. Electroanal. Chem..

[45] Yang Z., Tang Y., Li J., Zhang Y., Hu X. (2014). Facile synthesis of tetragonal columnar-shaped Tio_2_ nanorods for the construction of sensitive electrochemical glucose biosensor. Biosens. Bioelectron..

