# Supplementary material for: Facile Synthesis of β-Lactoglobulin-Functionalized Reduced Graphene Oxide and Trimetallic PtAuPd Nanocomposite for Electrochemical Sensing
Source: Nanomaterials (Basel). 2018 Sep 13;8(9):724. doi: 10.3390/nano8090724 (PMC6165462; doi:10.3390/nano8090724)
Supplement: Supplementary file 1 [file nanomaterials-08-00724-s001.pdf]

Supplementary

Facile Synthesis of β-Lactoglobulin-Functionalized Reduced Graphene Oxide and Trimetallic PtAuPd Nanocomposite for Electrochemical Sensing

Bingkai Han [1], Meixin Pan [1], Jiexin Zhou [1], Yingying Wang [1], Zihua Wang [1], Jun Jiao [1], Cong Zhang [2,*] and Qiang Chen [1,*]

[1] The Key Laboratory of Bioactive Materials Ministry of Education, College of Life Science, Nankai University, Weijin Road No. 94, Tianjin 300071, China; hanbingkai@mail.nankai.edu.cn (B.H.); 2120171090@mail.nankai.edu.cn (M.P.); zhoujiexin@mail.nankai.edu.cn (J.Z.); wangyingying@mail.nankai.edu.cn (Y.W.); zihuawang@mail.nankai.edu.cn (Z.W.); jyotika0201@gmail.com (J.J.)

[2] Department of Chemistry, School of Sciences, Hebei University of Science and Technology, Shijiazhuang 050018, China

* Correspondences: congzhang@hebust.edu.cn (C.Z.); qiangchen@nankai.edu.cn (Q.C.); Tel.: +86-022-2350-6122 (Q.C.)

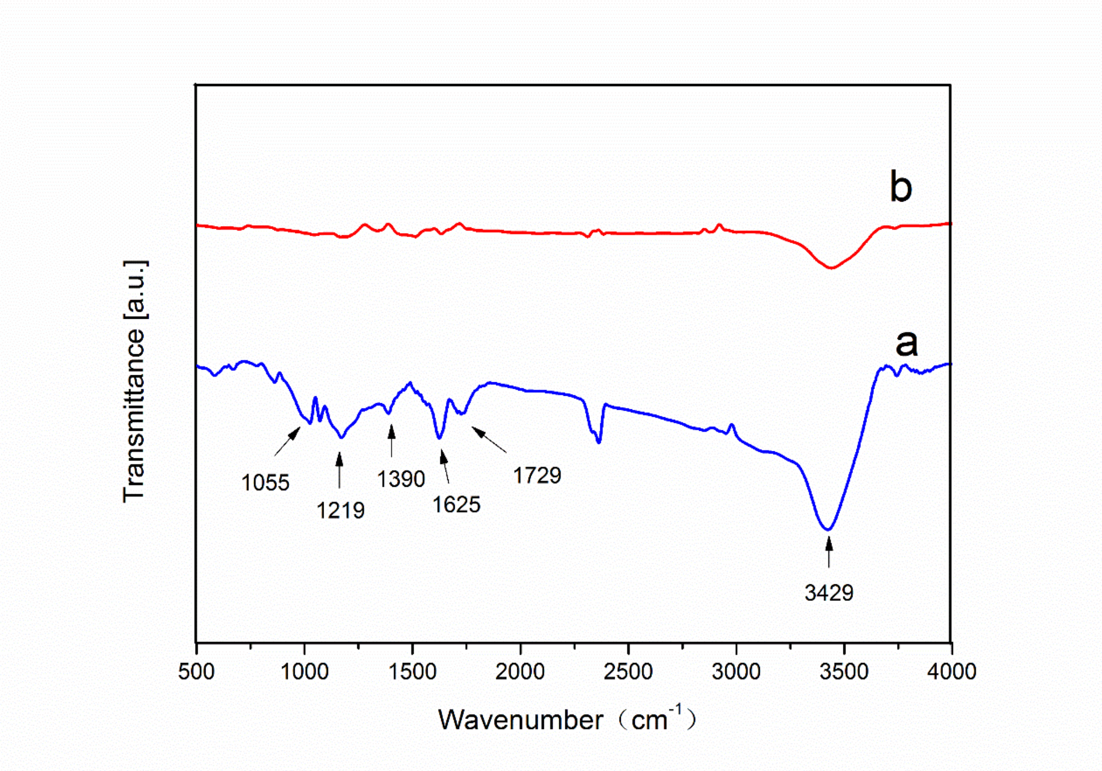

Figure S1. FT-IR patterns of GO (**a**) and BLG-PtAuPd-RGO (**b**).

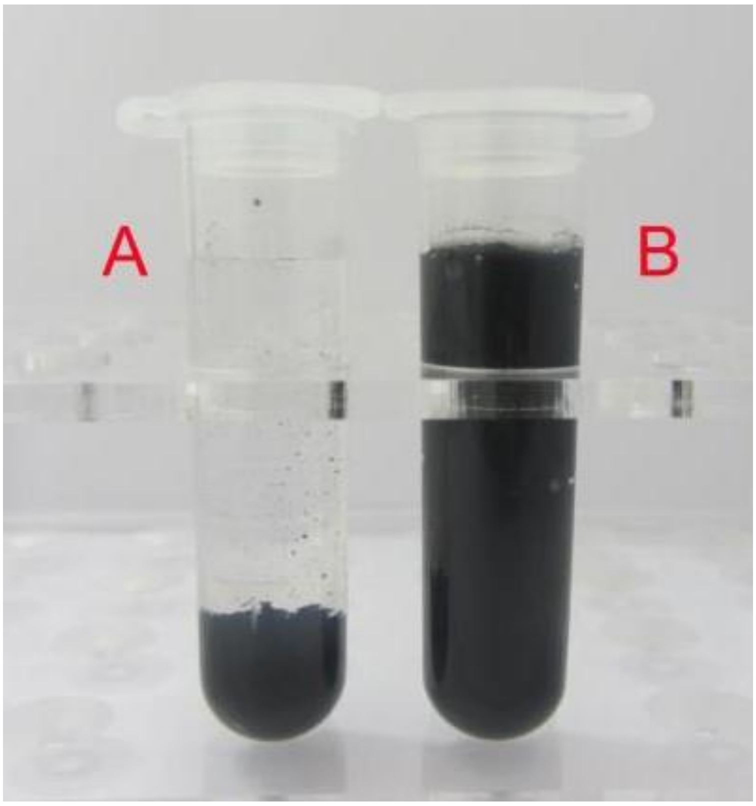

Figure S2. Synthetic composite materials with BLG (**B**) and without BLG (**A**) in a static position at indoor temperature for 48 hours.

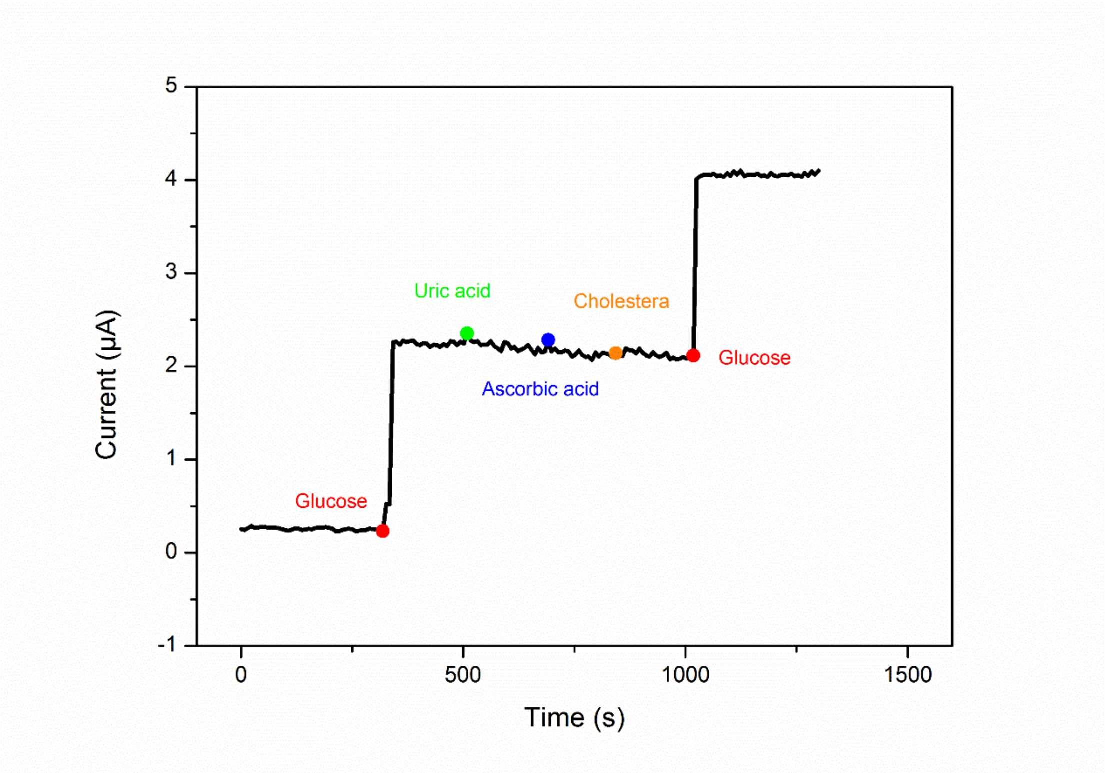

Figure S3. Amperometric response of GOD-BLG-PtAuPd-RGO/GC electrodes in 0.1 M PBS (PH 7.0) solution mixture at operating voltage of 600 mV with 0.5 mM glucose or other substance: uric acid (0.8 mM), ascorbic acid (0.6 mM) and cholesterol (0.6 mM).
